# Multicomponent new particle formation from sulfuric acid, ammonia, and biogenic vapors

**DOI:** 10.1126/sciadv.aau5363

**Published:** 2018-12-12

**Authors:** Katrianne Lehtipalo, Chao Yan, Lubna Dada, Federico Bianchi, Mao Xiao, Robert Wagner, Dominik Stolzenburg, Lauri R. Ahonen, Antonio Amorim, Andrea Baccarini, Paulus S. Bauer, Bernhard Baumgartner, Anton Bergen, Anne-Kathrin Bernhammer, Martin Breitenlechner, Sophia Brilke, Angela Buchholz, Stephany Buenrostro Mazon, Dexian Chen, Xuemeng Chen, Antonio Dias, Josef Dommen, Danielle C. Draper, Jonathan Duplissy, Mikael Ehn, Henning Finkenzeller, Lukas Fischer, Carla Frege, Claudia Fuchs, Olga Garmash, Hamish Gordon, Jani Hakala, Xucheng He, Liine Heikkinen, Martin Heinritzi, Johanna C. Helm, Victoria Hofbauer, Christopher R. Hoyle, Tuija Jokinen, Juha Kangasluoma, Veli-Matti Kerminen, Changhyuk Kim, Jasper Kirkby, Jenni Kontkanen, Andreas Kürten, Michael J. Lawler, Huajun Mai, Serge Mathot, Roy L. Mauldin, Ugo Molteni, Leonid Nichman, Wei Nie, Tuomo Nieminen, Andrea Ojdanic, Antti Onnela, Monica Passananti, Tuukka Petäjä, Felix Piel, Veronika Pospisilova, Lauriane L. J. Quéléver, Matti P. Rissanen, Clémence Rose, Nina Sarnela, Simon Schallhart, Simone Schuchmann, Kamalika Sengupta, Mario Simon, Mikko Sipilä, Christian Tauber, António Tomé, Jasmin Tröstl, Olli Väisänen, Alexander L. Vogel, Rainer Volkamer, Andrea C. Wagner, Mingyi Wang, Lena Weitz, Daniela Wimmer, Penglin Ye, Arttu Ylisirniö, Qiaozhi Zha, Kenneth S. Carslaw, Joachim Curtius, Neil M. Donahue, Richard C. Flagan, Armin Hansel, Ilona Riipinen, Annele Virtanen, Paul M. Winkler, Urs Baltensperger, Markku Kulmala, Douglas R. Worsnop

**Affiliations:** 1 Institute for Atmospheric and Earth System Research/Physics, Faculty of Science, University of Helsinki, P.O. Box 64, FI-00014 Helsinki, Finland.; 2 Laboratory of Atmospheric Chemistry, Paul Scherrer Institute, 5232 Villigen PSI, Switzerland.; 3 Finnish Meteorological Institute, Erik Palménin aukio 1, 00560 Helsinki, Finland.; 4 Faculty of Physics, University of Vienna, Boltzmanngasse 5, 1090 Wien, Austria.; 5 CENTRA and FCUL, Universidade de Lisboa, Campo Grande, 1749-016 Lisboa, Portugal.; 6 Goethe University Frankfurt, Institute for Atmospheric and Environmental Sciences, Altenhöferallee 1, 60438 Frankfurt am Main, Germany.; 7 University of Innsbruck, Institute for Ion and Applied Physics, 6020 Innsbruck, Austria.; 8 Ionicon GesmbH, Innsbruck, Austria.; 9 University of Eastern Finland, Department of Applied Physics, P.O. Box 1627, 70211 Kuopio, Finland.; 10 Carnegie Mellon University Center for Atmospheric Particle Studies, 5000 Forbes Avenue, Pittsburgh, PA 15213, USA.; 11 Department of Chemistry, University of California, Irvine, Irvine, CA 92697, USA.; 12 Department of Chemistry and CIRES, University of Colorado, Boulder, CO 80309 USA.; 13 University of Leeds, Leeds LS2 9JT, UK.; 14 Aerosol and Haze Laboratory, Beijing University of Chemical Technology, Beijing, China.; 15 California Institute of Technology, 210-41, Pasadena, CA 91125, USA.; 16 CERN, CH-1211 Geneva, Switzerland.; 17 Department of Environmental Science and Analytical Chemistry (ACES) and Bolin Centre for Climate Research, Stockholm University, 10691 Stockholm, Sweden.; 18 School of Earth and Environmental Sciences, University of Manchester, Manchester M13 9PL, UK.; 19 Joint International Research Laboratory of Atmospheric and Earth System Sciences, Nanjing University, Nanjing, China.; 20 Collaborative Innovation Center of Climate Change, Jiangsu Province, China.; 21 IDL, Universidade da Beira Interior, Covilhã, Portugal.; 22 Laboratory of Environmental Chemistry, Paul Scherrer Institute, 5232 Villigen PSI, Switzerland.; 23 Aerodyne Research Inc., 45 Manning Road, Billerica, MA 01821, USA.; 24 Aerosol Physics, Faculty of Science, Tampere University of Technology, P.O. Box 692, 33101, Tampere, Finland.; 25 Helsinki Institute of Physics, FI-00014 Helsinki, Finland.

## Abstract

A major fraction of atmospheric aerosol particles, which affect both air quality and climate, form from gaseous precursors in the atmosphere. Highly oxygenated organic molecules (HOMs), formed by oxidation of biogenic volatile organic compounds, are known to participate in particle formation and growth. However, it is not well understood how they interact with atmospheric pollutants, such as nitrogen oxides (NO_*x*_) and sulfur oxides (SO_*x*_) from fossil fuel combustion, as well as ammonia (NH_3_) from livestock and fertilizers. Here, we show how NO_*x*_ suppresses particle formation, while HOMs, sulfuric acid, and NH_3_ have a synergistic enhancing effect on particle formation. We postulate a novel mechanism, involving HOMs, sulfuric acid, and ammonia, which is able to closely reproduce observations of particle formation and growth in daytime boreal forest and similar environments. The findings elucidate the complex interactions between biogenic and anthropogenic vapors in the atmospheric aerosol system.

## Teaser

Atmospheric aerosol formation from biogenic vapors is strongly affected by air pollutants, like NO_*x*_, SO_2_, and NH_3_.

## INTRODUCTION

Atmospheric new particle formation (NPF) can dominate regional concentrations of aerosol particles and cloud condensation nuclei (CCN) and significantly contribute to their global budgets (*1*–*3*). Because variations in CCN concentrations affect aerosol-cloud interactions and associated climate forcing, it is vital to understand both past changes to CCN since the industrial revolution and also expected future changes, as emissions from fossil fuel combustion decline in response to efforts to improve air quality and mitigate climate change (*4*).

NPF begins with the formation of molecular clusters from low-volatility vapors and continues with their subsequent growth to aerosol particles under favorable conditions (*5*, *6*). Sulfuric acid is believed to govern NPF in most environments, although it cannot alone explain the observed formation and growth rates (GRs) (*7*, *8*). Particle growth, on the other hand, has been closely linked to organic vapors (*9*), which are abundant in the continental boundary layers. Highly oxygenated organic molecules (HOMs) with exceedingly low vapor pressures can be involved at the very early stages of particle formation (*10*–*12*), but very few field studies have unambiguously observed NPF without sulfuric acid (*13*, *14*). Despite numerous laboratory and field studies, interactions between organic and inorganic constituents, as well as their relative roles in atmospheric NPF, remain highly uncertain. It is also crucial to resolve whether the strong enhancement of nucleation rates by ions, which was observed in the pure systems (*15*, *16*), occurs also when organic vapors interact with other compounds.

Recent laboratory experiments with comprehensive instrumentation and low contaminant levels have shown how NPF can proceed via a binary mechanism (water and sulfuric acid) (*16*–*18*), a ternary inorganic mechanism (water, sulfuric acid, and base) (*16*, *19*–*21*), or a ternary organic mechanism (water, sulfuric acid, and organics) (*10*, *11*, *22*) or by nucleation of HOMs alone, i.e., pure biogenic nucleation (*15*). These experiments have constrained the particle formation rates in these model systems; however, none of them have reproduced conditions of the daytime atmospheric boundary layer, especially the boreal forest where NPF is very common (*5*). Some of the main differences are that most of the previous laboratory experiments did not include NO_*x*_ or they did not control the NH_3_ concentrations.

NO_*x*_ influences organic oxidation indirectly by changing the oxidant balance (OH versus ozone and NO_3_) and directly by perturbing oxidation mechanisms, especially the branching of peroxy radical (RO_2_) reactions, which is crucial in the production of HOMs. NO_*x*_ can decrease yields of secondary organic aerosol (SOA) (*23*, *24*) and suppress NPF from terpenes (*25*), possibly by shutting off RO_2_ autoxidation leading to HOMs (*12*) and, instead, forming (relatively) more volatile organonitrates (ONs) (*23*). The oxidation of SO_2_, on the other hand, leads to the formation of sulfuric acid, which has a very low vapor pressure. Sulfuric acid also clusters very efficiently with bases (*19*), but whether this happens in the presence of organics is not known until now. Thus, both enhancement and suppression of NPF by human activity is possible, depending on conditions.

## RESULTS

To simulate NPF and growth under realistic daytime conditions resembling those in the boreal forest (our reference being the Hyytiälä SMEAR II station in southern Finland), we performed experiments in the CLOUD (Cosmics Leaving OUtdoors Droplets) chamber at CERN (European Organization for Nuclear Research). All experiments were performed at 278 K and 38% relative humidity (RH) and included monoterpenes (MTs; C_10_H_16_). We used a 2:1 volume mixture of alpha-pinene and delta-3-carene, which are the two most abundant MTs in Hyytiälä (*26*). The ozone mixing ratio in the chamber was ca. 40 parts per billion by volume (ppbv), and the hydroxyl radical (OH) concentration was controlled with an ultraviolet (UV) light system (see Materials and Methods). We first performed experiments without SO_2_ (H_2_SO_4_ concentration of <2 × 10^5^ cm^−3^) and then added 0.5 to 5 ppbv of SO_2_, leading to 1 × 10^6^ to 7 × 10^7^ cm^−3^ of H_2_SO_4_ in the chamber. The experiments were conducted with various mixing ratios of NO_*x*_ (=NO + NO_2_, 0 to 5 ppbv) and ammonia [2 to 3000 parts per trillion by volume (pptv)], covering the range from very clean to polluted environments. Most experiments were first performed without ions in the chamber (neutral conditions, N) and then repeated with ionization from galactic cosmic rays (GCR conditions).

**Fig. 1:**
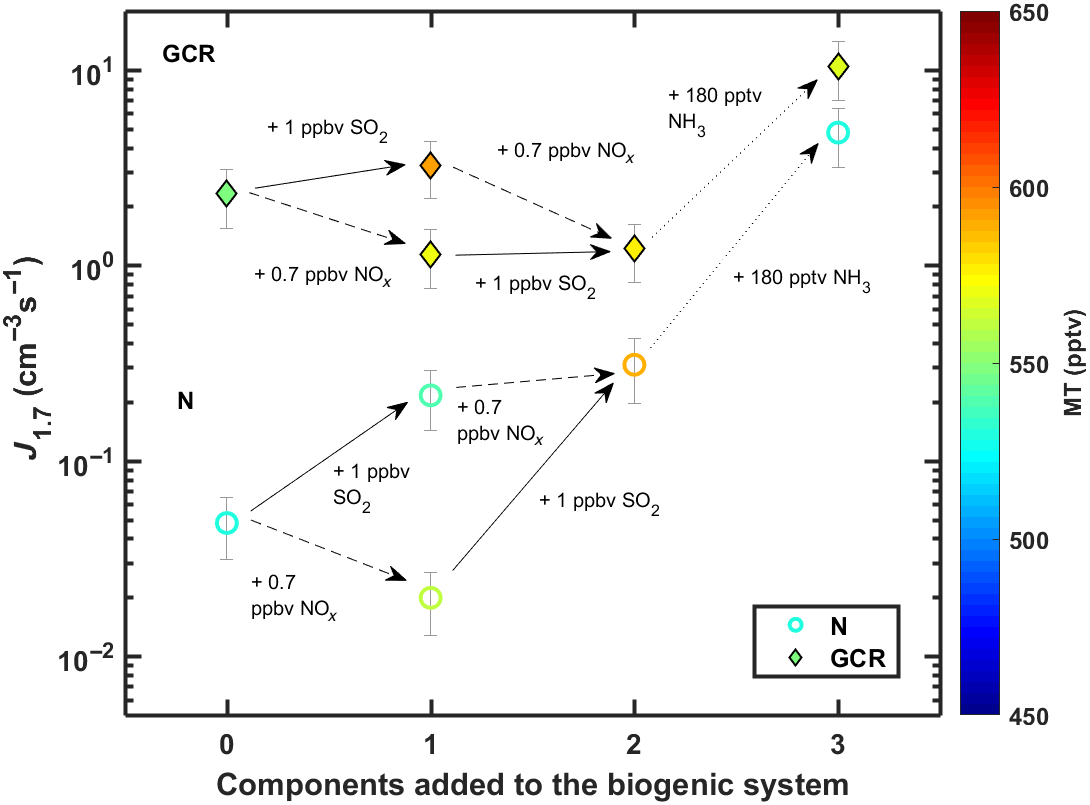
The effect of adding different vapors on biogenic nucleation rates (*J*_1.7_). All points have similar MT (530 to 590 pptv) and ozone (40 ppbv) mixing ratios. The leftmost points were measured with only MTs added to the chamber, and each step to the right represents addition of one more component to the system. Solid arrows describe the addition of ca. 1 ppbv of SO_2_ (resulting in an H_2_SO_4_ concentration of 1 × 10^7^ to 2 × 10^7^ cm^−3^), dashed arrows describe the addition of ca. 0.7 ppbv of NO_*x*_, and dotted arrows describe the addition of ca. 180 pptv of NH_3_. Circles are experiments at neutral conditions (N), and diamonds are experiments at GCR conditions. Colors of the symbols indicate the measured MT mixing ratio. The error bars describe the uncertainty in the nucleation rates, which was calculated similar to earlier CLOUD publications, taking into account both the systematic and statistical errors and run-to-run repeatability (see Supplementary Materials and Methods). See fig. S1 for the formation rate of 2.5-nm particles.

Figure 1 shows the step-by-step change in nucleation rates (*J*) when going from a single-component system toward a more realistic multicomponent mixture. Compared to the pure biogenic system with only MTs in the chamber, fewer new particles are formed when NO_*x*_ is added and more particles are formed when SO_2_ is added (Fig. 1 and figs. S1 and S2). A further increase is observed when ammonia is added to the chamber as well. To understand the mechanism and magnitude of these effects, we will first discuss the reduction of particle formation by NO_*x*_ and then the increase by addition of SO_2_ and NH_3_ and finally show how each of these compounds are needed to explain NPF and growth in the multicomponent system.

### Effect of NO_*x*_ on particle formation rates

We find that the particle formation rates largely follow the ratio of MT to NO_*x*_ in the chamber (fig. S3), as reported in an earlier study, albeit for larger particles (*25*). However, to discover the underlying cause of this pattern, we need to understand what happens to HOMs when NO_*x*_ is added to the chamber. Increasing the NO_*x*_ concentration leads to a larger fraction of ONs among all HOMs and a significant decrease in dimers, although the total HOM concentration slightly increases. Therefore, the volatility distribution is shifted toward more volatile products. This is consistent with lower SOA mass yields from terpenes at high NO_*x*_ concentrations (*23*, *24*).

**Fig. 2:**
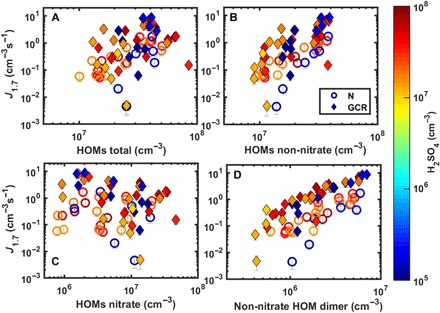
Relation of nucleation rates to different HOM categories. Nucleation rates (*J*_1.7_) as a function of the (**A**) total concentration of HOMs [regardless whether the molecule has nitrate group(s) or not], (**B**) non-nitrate HOMs, (**C**) nitrate HOMs (ONs), and (**D**) non-nitrate HOM dimers. Open circles refer to neutral experiments, closed diamonds refer to GCR experiments, and the color refers to the H_2_SO_4_ concentration (blue points were measured without added SO_2_). All points were measured at 278 K and 38% RH, with varying MT concentrations (100 to 1500 pptv) and NO_*x*_ levels (0 to 5 ppbv; NO/NO_2_, about 0.6%) without added NH_3_.

In contrast to pure biogenic experiments (*15*), the nucleation rates in the presence of NO_*x*_ do not correlate with the total HOM concentration (Fig. 2A). Therefore, we further divided the HOMs into four groups: non-nitrate HOM monomers (C_4–10_H_*x*_O_*y*_), non-nitrate HOM dimers (C_11–20_H_*x*_O_*y*_), ON monomers (C_4–10_H_*x*_O_*y*_N_1–2_), and ON dimers (C_11–20_H_*x*_O_*y*_N_1–2_). We find a clear difference in how non-nitrate HOMs and ONs relate to the nucleation rates (Fig. 2 and table S1). The nucleation rates correlate with non-nitrate HOMs (Pearson’s correlation coefficient *R* = 0.72 for GCR experiments), especially with dimers (*R* = 0.97), but not with ONs (*R* = −0.42).

It should be noted that the effect of NO_*x*_ chemistry on HOM formation, and the subsequent NPF, might depend on the organic molecule in question; alpha-pinene has been reported to behave differently with respect to SOA formation than some other MTs and sesquiterpenes (*24*, *27*). For any given volatile organic compound (VOC) concentration, the HOM yield and volatility distribution, both of which are altered by NO_*x*_, matter for the NPF efficiency. Our results are specific to photo-oxidation, i.e., daytime conditions.

### Effect of SO_2_ and NH_3_ on particle formation rates

Let us next consider the addition of SO_2_, which quickly forms H_2_SO_4_ in the chamber by OH oxidation under the presence of UV light. Without added ammonia (background NH_3_ estimated to be ca. 2 pptv), *J* shows no correlation with sulfuric acid (*R* = −0.06; table S1), consistent with an earlier CLOUD observation (*15*) that H_2_SO_4_ does not affect nucleation from alpha-pinene ozonolysis at H_2_SO_4_ < 6 × 10^6^ cm^−3^. Our experiments with somewhat higher sulfuric acid concentration (H_2_SO_4_ ≥ 1 × 10^7^ cm^−3^) show consistently slightly higher *J* at the same HOM concentration than the experiments without SO_2_ (Figs. 1 and 2D). At low HOM dimer concentrations, the pure biogenic *J* drops below the detection threshold, although particle formation could still be observed together with H_2_SO_4_ (Fig. 2D). This indicates that H_2_SO_4_ is able to interact with HOMs to form particles, as speculated earlier (*11*), but the mechanism is inefficient without NH_3_ (or another base).

**Fig. 3:**
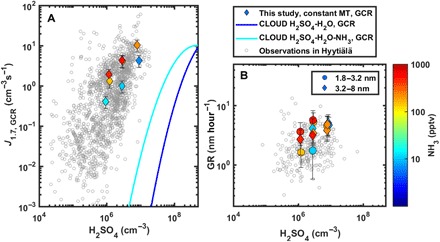
Nucleation and GRs at CLOUD compared to atmospheric observations in Hyytiälä. Here, we chose a series of experiments with constant MT/NO_*x*_ ratio (ca. 0.6, NO/NO_2_ = 7%), while H_2_SO_4_ and NH_3_ concentrations were varied across the range relevant for boreal forest. (**A**) Nucleation rates (*J*_1.7_) at CLOUD (colored points) and ambient observations in Hyytiälä (*5*, *8*) (gray circles). The blue and cyan lines represent binary (H_2_SO_4_-H_2_O) and ternary (H_2_SO_4_-H_2_O-NH_3_, 7 < [NH_3_] < 40 pptv) nucleation, respectively, based on earlier CLOUD data (*21*), while the pure biogenic nucleation rate at similar MT/NO_*x*_ ratio would be <1 cm^−3^ s^−1^ (fig. S3). (**B**) GRs of 1.8- to 3.2-nm-sized and 3.2- to 8-nm-sized particles in the same experiments compared to observations of initial GR in Hyytiälä (*40*).

Ammonia strongly enhances nucleation rates (Fig. 1 and figs. S1, S2, and S4) when both H_2_SO_4_ and HOMs are present simultaneously. In general, experiments at higher NH_3_ (≥200 pptv) show up to two orders of magnitude higher *J* than otherwise similar experiments without added NH_3_ (Fig. 1 and fig. S4). The multicomponent experiments with all three precursors—MT, H_2_SO_4_, and NH_3_—in the presence of NO_*x*_ are able to qualitatively and quantitatively reproduce boreal forest nucleation and GRs (Fig. 3). The ternary inorganic mechanism (H_2_SO_4_, NH_3_, and water) cannot explain them, as it produces very few particles at H_2_SO_4_ concentrations below 1 × 10^7^ cm^−3^ and temperatures of ≥278 K (*16*, *21*), although most NPF events in Hyytiälä occur at these conditions (Fig. 3A). The pure biogenic mechanism, on the other hand, does not show a similar H_2_SO_4_ dependency as observed in the atmosphere, and it produces significant nucleation rates (*J ≥* 1 cm^−3^ s^−1^) only without NO_*x*_ or when NO_*x*_ is low compared to MT concentrations (MT/NO_*x*_ ≥ 1) (fig. S3). Thus, the nucleation rates detected during multicomponent experiments cannot be explained solely by the sum of ternary inorganic and pure biogenic nucleation (Fig. 3A).

### Particle formation and growth in multicomponent experiments

Combining the observations listed above, we postulate that the formation rates in the multicomponent system can be parametrized with the empirical formula

$$J=k_{1}{\left[H_{2}{\text{SO}}_{4}\right]}^{a}{\left[{\text{NH}}_{3}\right]}^{b}{\left[{\text{HOM}}_{\text{di}}\right]}^{c}$$ (1)

where [HOM_di_] is the concentration of non-nitrate HOM dimers and *k*_1_, *a*, *b*, and *c* are free parameters. This approach builds on the many observations showing that measured nucleation rates in the continental boundary layer seem to follow a power-law functional dependency on sulfuric acid concentration

$$J=k{\left[H_{2}{\text{SO}}_{4}\right]}^{p}$$ (2)

with the exponent *p* varying between 1 and 2 (*6*–*8*). The prefactor *k* varies considerably between different locations, as it includes the variation of nucleation rates due to external conditions (*T*, RH, etc.) and any conucleating vapors. On the basis of earlier CLOUD data showing the participation of oxidized organics in the first steps of particle formation (*11*), the parametrization was rewritten as

$$J=k_{2}{\left[H_{2}{\text{SO}}_{4}\right]}^{p}{\left[\text{BioOxOrg}\right]}^{q}$$ (3)

Compared to Eq. 3, we have now included a dependency on ammonia and further defined the oxidized organics participating in particle formation to be mainly non-nitrate HOM dimers. In the next section, we will show that all of these species can participate in clustering simultaneously.

**Fig. 4:**
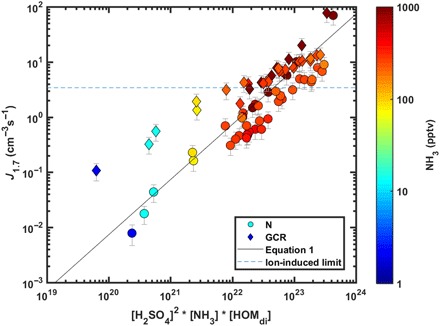
Nucleation rates (*J*_1.7_) as a function of the product of the concentrations of H_2_SO_4_, NH_3_, and non-nitrate HOM dimers. Circles refer to neutral experiments, diamonds refer to GCR experiments, and the color refers to the NH_3_ concentration. All points here were measured at 278 K and 38% RH. The MT mixing ratio was varied between 100 and 1200 pptv, H_2_SO_4_ concentration between 5 × 10^6^ and 6 × 10^7^ cm^−3^, NH_3_ between 2 and 3000 pptv, and NO_*x*_ between 0.7 and 2.1 ppbv (NO/NO_2_ = 0.6%). The dashed line gives the maximum rate from ion-induced nucleation based on the ion pair production rate in CLOUD under GCR conditions (*15*). The solid line is the multicomponent parametrization for neutral experiments based on Eq. 1 with *k* = 7.4 × 10^−23^ s^−1^ pptv^−1^ cm^6^.

Using Eq. 1 with *a* = 2, *b* = *c* = 1, we can find an extremely good correlation (*R* = 0.96) between the modeled and measured formation rates for the set of neutral experiments at 10 < NH_3_ < 3000 pptv, 5 × 10^6^ < H_2_SO_4_ < 6 × 10^7^ cm^−3^, 100 < MT < 1200 pptv, 0.7 < NO_*x*_ < 2.1 ppbv, and O_3_ = 40 ppbv (Fig. 4 and fig. S5). Replacing [HOM_di_] with [MT/NO_*x*_] still gives a high correlation (*R* = 0.92). However, using Eq. 3 with *p* = 2, *q* = 1 as in (*11*) and [BioOxOrg] = [HOMs], the correlation is worse, *R* = 0.53, mainly due to varying NO_*x*_ and NH_3_ concentrations not included in the earlier parametrization (fig. S5). A more sophisticated multicomponent parametrization, which can be extended to a larger set of conditions (*T*, RH, ion concentration, etc.) and a wider range of vapor concentrations, is subject to future studies.

The enhancement of *J* due to ions decreases with increasing NH_3_ concentration and *J* (Fig. 4 and fig. S4) and is generally considerably weaker in the multicomponent system than in the acid-base or pure biogenic systems (*15*, *16*) at otherwise similar vapor concentrations (Fig. 1). This means that the neutral nucleation pathway is more efficient in the multicomponent system. In general, ion enhancement becomes weaker with increasing stability of the forming neutral clusters, indicating that chemical interactions between different kinds of molecules become more important in cluster bonding. This might, at least partly, explain why field studies have found only minor contribution of ions to NPF in various environments (*5*, *13*, *28*), as multiple vapors are always present in the atmosphere.

The formation rate is not the only important factor governing NPF. The competition between the GR of newly formed particles and their loss rate governs the fraction of particles that eventually reach CCN sizes. Because particle losses are most severe in the beginning of the growth process, initial GRs in the sub–3-nm size range are especially critical (*29*). Particle GRs in our experiments, over the same ranges of gas concentrations as above, seem to follow a formula

$$\text{GR}\;=k_{1}{\left[H_{2}{\text{SO}}_{4}\right]}^{a}+k_{2}{\left[H_{2}{\text{SO}}_{4}\right]}^{b}{\left[{\text{NH}}_{3}\right]}^{c}+k_{3}{\left[\text{Org}\right]}^{d}$$ (4)

where the first term can be interpreted as growth by condensation of sulfuric acid (*30*), the second term by sulfuric acid ammonia clusters (*31*), and the third term by oxidized organics (*32*). As we concentrate on the initial GRs, we chose [Org] to include only non-nitrate HOM dimers, which are the most relevant in this size range (<7 nm). Again, taking *a* = *b* = *c* = *d* = 1, we find a very good correlation especially for the size range 3.5 to 7 nm (*R* = 0.94) between modeled and measured GRs (fig. S6). It should be noted that the coefficients *k* are size dependent and, especially, that for different size ranges a different subset of organic vapors is relevant for growth (*32*). As the particles grow, a wider range of vapors with different volatilities can contribute to the growth, and the third term grows progressively more important (fig. S6). This conforms to the present qualitative picture of the particle growth process in the boreal forest (*5*), and the measured values are in the same order of magnitude as those observed in Hyytiälä (Fig. 3B).

Here, we assume no interaction between organics and sulfuric acid or organics and ammonia in particle growth, which could be relevant in other conditions. However, when using measured sulfuric acid concentrations, we cannot accurately model the GRs without a term depending on NH_3_ concentrations. This is consistent with the recent findings that bases can enhance initial GRs (*31*, *33*), e.g., due to a significant fraction of sulfuric acid bonded to acid-base clusters (*31*, *34*) and therefore not included in the sulfuric acid monomer measurement. It should be noted that reactive uptake, particle-phase reactions, and other growth mechanisms than nonreversible condensation can be important for growth at larger sizes.

### Composition of clusters during multicomponent experiments

**Fig. 5:**
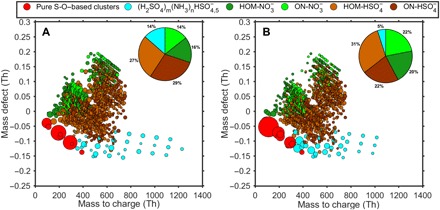
Negative ions and ion clusters detected during multicomponent NPF in the CLOUD chamber and in Hyytiälä. The mass defect shows the difference between nominal and exact mass of the ions detected with the negative atmospheric pressure interface–time-of-flight mass spectrometer. (**A**). Data from the CLOUD chamber, averaged over several experiments (the orange and red points in Fig. 3) with H_2_SO_4_ (1 × 10^6^ to 1 × 10^7^ cm^−3^), NO_*x*_ (1 ppb), and NH_3_ (200 to 500 pptv). (**B**) Data from Hyytiälä during an NPF event on 5 April 2012. The colored symbols indicate the identified ions: pure sulfuric acid and S-O–based clusters (red), sulfuric acid–ammonia clusters (cyan), HOMs clustered with NO_3_^−^ (dark green), ONs clustered with NO_3_^−^ (light green), HOMs clustered with HSO_4_^−^ (light brown), and ON clustered with HSO_4_^−^ (dark brown). The symbol size corresponds to the relative signal intensity on a logarithmic scale. The pie charts give the fraction of all identified peaks, excluding the pure S-O–based peaks.

We measured the chemical composition of freshly formed clusters with mass spectrometric methods, shown as a mass defect plot (Fig. 5A and fig. S7). The mass spectra from the multicomponent experiments are remarkably similar to those recorded in Hyytiälä during NPF (Fig. 5B) (*10*, *35*), indicating that the underlying chemistry in the chamber was very similar to that under ambient atmospheric conditions.

We find that HOMs, H_2_SO_4_, and NH_3_ are able to cluster with each other in many different ways. Similar to pure biogenic experiments (*15*), we detect non-nitrate HOMs clustered with NO_3_^−^; but now we detect also ONs clustered with NO_3_^−^. Both non-nitrate HOMs and ONs are also capable of forming clusters with HSO_4_^−^. While the upper part of the mass defect plot (Fig. 5) is characterized by these organic clusters, the lower part is dominated by inorganic clusters. In addition to pure sulfuric acid clusters [(H_2_SO_4_)_0–3_HSO_4,5_^−^)], we see sulfuric acid clusters containing ammonia, the largest one being (H_2_SO_4_)_9_(NH_3_)_8_HSO_4_^−^. During ternary (H_2_SO_4_-H_2_O-NH_3_) nucleation, the entire spectrum is composed solely of those two compounds, up to 1500 Thomson (Th), with approximately one-to-one acid-base ratio (*10*). However, this is not the case in the multicomponent experiments or in the atmosphere. We believe that, once larger acid-base clusters are formed, they can interact with organics, creating very large clusters, whose identities cannot be resolved with current instrumentation due to their size and complex elemental composition. Some multicomponent HOM-H_2_SO_4_-NH_3_-NH_4_^+^ clusters can be detected in the positive ion side. Positive ions are mainly composed of non-nitrate HOMs and ONs up to tetramer, with and without ammonia as core ion, and H_2_SO_4_-NH_3_-NH_4_^+^ clusters (fig. S7). The clusters might also contain water molecules that evaporate during sampling.

## DISCUSSION

In summary, we have shown that sulfuric acid, ammonia, and organic vapors have a synergetic effect on NPF. Sulfuric acid, together with ammonia, can enhance particle formation in situations when the HOM concentration alone is not high enough to form substantial amounts of particles and enables the formed particles to grow past 3 nm before the biogenic vapors take over in the growth process. The efficiency of biogenic vapors to form aerosol particles strongly depends on the amount of non-nitrate HOMs formed; thus, higher NO_*x*_ concentrations tend to suppress NPF and initial growth in environments similar to daytime boreal forest, while the growth of larger particles is less severely affected. Nucleation and GRs are sensitive to changes in any of the precursor vapor concentrations (HOMs, H_2_SO_4_, and NH_3_) and the NO_*x*_ concentration. This sensitivity can partly explain the wide range of observed atmospheric nucleation rates for a given sulfuric acid concentration.

We have measured three critical parameters associated with NPF: the nucleation rate, the GR, and the composition of the growing clusters. All three are consistent with observations in the atmosphere. Thus, we are able to reproduce the observations at daytime boreal forest conditions in the laboratory. The results from a chemical transport model (fig. S8) show that there is almost always sufficient NH_3_ in the continental boundary layer to combine efficiently with H_2_SO_4_ and HOMs due to effective long-range transport of anthropogenic pollutants. This pattern favors the multicomponent mechanism over pure biogenic nucleation in the present-day atmosphere. The results presented here can almost certainly be extended to other chemical systems; specifically, HOMs can be produced from other organic vapors than MTs, and the stabilizing agent for sulfuric acid could be amines in addition to ammonia. Therefore, we believe that the multicomponent acid-base organic mechanism is dominant in the continental boundary layer in all relatively clean to moderately polluted present-day environments.

Possible future reductions in anthropogenic emissions of SO_2_ and NH_3_ may reduce particle formation involving H_2_SO_4_, while a reduction of NO_*x*_ could possibly promote NPF from organic vapors. Thus, the climate effects of these measures depend strongly on which compounds are regulated. Understanding the complex interplay between different anthropogenic and biogenic vapors, their oxidants, and primary particles remains a key question in assessing the role of NPF in the global climate system.

## MATERIALS AND METHODS

### Experimental design

The objective of this study was to explore the conditions required to replicate daytime NPF and growth as it is observed at the Hyytiälä SMEAR II station, which is one of the most studied field sites in this respect, located in the boreal forest region in southern Finland (*36*). Most of the experiments were performed during September to December 2015 (CLOUD10 campaign) at the CLOUD facility (see below) at CERN, Geneva. To find the correct combination of condensable vapors, we first measured nucleation and GRs in the presence of pure biogenic precursors only (mixture of alpha-pinene and delta-3-carene). The total MT mixing ratio was varied between 100 and 1500 pptv. The background sulfuric acid concentration for those experiments was <2 × 10^5^ cm^−3^. Then, 1 to 5 ppbv of SO_2_ were added to study the influence of sulfuric acid on pure biogenic nucleation, resulting in sulfuric acid concentrations of 5 × 10^6^ to 6 × 10^7^ cm^−3^. The measurements at different SO_2_-MT concentration pairs were repeated at four different mixing ratios of nitrogen oxides in the chamber 0, 0.7, 2, and 5 ppbv, with a NO/NO_2_ ratio of ca. 0.6%. Here, we aimed to produce a similar fraction of ONs from all HOMs, as is observed in Hyytiälä during NPF. Last, we added ammonia (10 to 3000 pptv) to the chamber and repeated a subset of experiments in the presence of all the precursors (MTs, SO_2_, and NH_3_) and NO_*x*_. The estimated background NH_3_ mixing ratio in the chamber (i.e., before NH_3_ addition) is ca. 2 pptv (*21*, *37*).

In fall 2016, additional experiments were performed during the CLOUD11 campaign at lower H_2_SO_4_ concentrations (1 × 10^6^ to 2 × 10^7^ cm^−3^), two MT mixing ratios (600 and 1200 pptv), and three NH_3_ levels (~10, 200, and 500 pptv). Between CLOUD10 and CLOUD11 campaigns, the UV light system in the chamber was enhanced (see below), enabling using a 7% NO/NO_2_ ratio with 1 ppbv of total NO_*x*_, typical of daytime Hyytiälä (*38*). Figures 3 and 5 and fig. S7 show data from the CLOUD11 campaign. Although the relation between *J* and HOMs and H_2_SO_4_ and NH_3_ was explored at a NO/NO_2_ ratio lower than 7% (Figs. 1, 2, and 4), we believe that this affects mainly the fraction of non-nitrate to nitrate HOMs in the chamber and not the particle formation process from the product molecules.

To study the neutral and ion-induced nucleation pathway separately, most of the experiments were conducted first at neutral and then at GCR (see below) conditions. All of the experiments for this study were performed at 278 K and 38% RH.

It should be noted that our current study differs in several important ways from Riccobono *et al*. (*11*) and Schobesberger *et al*. (*10*), which also show quantitative agreement of the nucleation rates from a chamber study with ambient observations, in the absence of added NH_3_. First, and most importantly, the experiments in those studies focused on second-generation products formed via oxidation of pinanediol, a very low vapor pressure surrogate for first-generation alpha-pinene oxidation products, so the chemical system was different. The SOA mass yields from pinanediol are much higher than those from alpha-pinene itself, and it is plausible that the oxidation products require less stabilization than the first-generation products studied here. Second, those experiments did not include NO_*x*_, which at least partly compensates the enhancing effect from NH_3_. Moreover, the mass spectra in the study of Riccobono *et al*. (*11*) revealed some clusters including NH_3_ and dimethylamine at the low pptv level. Further experiments would be required to assess the enhancement of *J* by trace concentrations of amines in a HOM-H_2_SO_4_ system.

### The CLOUD facility

The CLOUD chamber (*16*, *17*) is a temperature-controlled stainless steel cylinder with a volume of 26.1 m^3^ located at CERN, Geneva, Switzerland. To ensure cleanliness, all inner surfaces of the chamber are electropolished. Before each campaign, the chamber was rinsed with ultrapure water and subsequently heated to 373 K. While cooling down to operating temperature, the chamber was flushed with humidified synthetic air containing several ppmv (parts per million by volume) of ozone. Thus, the background total VOC concentration is in the sub-ppbv level (*39*) and the contamination from condensable vapors is mostly below the detection limit of our instruments [sub-pptv (*15*)]. A sophisticated gas supply system was used to carefully control the amounts of trace gases added to the chamber.

A high voltage field cage (±30 kV) inside the chamber can be switched on to remove all ions from the chamber (referred to as “neutral conditions,” N). When the electric field is off, natural GCRs are creating ions in the chamber, as is the situation in the atmosphere. This is referred to as “GCR conditions.” Ion concentrations in the chamber can be artificially increased by using the pion beam from the CERN Proton Synchrotron (3.5 GeV/c). This is called “π conditions” (not used in this study).

The chamber was equipped with several UV light systems. In all the experiments described in this study, so-called UVH light (4 × 200 W Hamamatsu Hg-Xe lamps producing light in the wavelength range of 250 to 450 nm) was used to produce OH. In CLOUD10, additionally, a UV laser (4-W excimer laser; KrF, 248 nm) was used in some of the experiments to achieve higher H_2_SO_4_ concentrations. Between the CLOUD10 and CLOUD11 campaigns, the intensity of the UVH light was increased by renewing and shortening the optical fibers, which deliver the light into the chamber. Therefore, the use of the UV laser was not necessary, as the UVH system could supply the same wavelengths. In CLOUD11, also a UV-sabre (400-W UVS3, centered on 385 nm) was available, with the main purpose to form NO from NO_2_. Thus, the NO/NO_2_ ratio could be controlled by changing the UV-sabre light intensity. The NO_2_ photolysis frequency, *j*_NO2_, was characterized using NO_2_ actinometry and varying the UV-sabre intensity. In CLOUD10, we injected NO directly into the chamber (leading to a constant NO/NO_2_). More details of the facility can be found elsewhere (*16*, *17*).

The instruments used to record chamber conditions, gas and particle concentration, as well as methods to calculate particle formation and GRs were similar to previous CLOUD publications, and they are described in Supplementary Materials and Methods.

### Statistical analysis

The correlation coefficients mentioned in the text and some figure captions were calculated with Matlab using function corrcoef, which gives Pearson’s correlation coefficient and the associated *P* values for testing the null hypothesis that there is no relationship between the observed phenomena. The correlation is considered significant when *P* is smaller than 0.05. The correlation coefficients, *P* values, and sample sizes between the nucleation rates (*J*_1.7_) and different gas phase precursor concentrations are summarized in table S1 separately for neutral and GCR experiments before and after NH_3_ addition.

## Supplementary Material

http://advances.sciencemag.org/cgi/content/full/4/12/eaau5363/DC1

## SUPPLEMENTARY MATERIALS

Supplementary material for this article is available at http://advances.sciencemag.org/cgi/content/full/4/12/eaau5363/DC1 Supplementary Materials and Methods Fig. S1. The effect of different additional vapors on the NPF rates (*J*_2.5_). Fig. S2. The effect of different additional vapors on the biogenic nucleation rate (*J*_1.7_) at different NO_*x*_ concentrations. Fig. S3. Nucleation rates (*J*_1.7_) as a function of the MT to NO_*x*_ ratio (MT/NO_*x*_). Fig. S4. Nucleation rates (*J*_1.7_) as a function of NH_3_ mixing ratio. Fig. S5. Modeled versus measured nucleation rates. Fig. S6. Modeled versus measured GRs. Fig. S7. Positive ions and ion clusters detected during multicomponent NPF in the CLOUD chamber. Fig. S8. Global annual mean concentrations of vapors involved in NPF. Table S1. Pearson’s correlation coefficient (*R*) between *J*_1.7_ and the concentration of different precursors in the chamber. References (*41*–*56*)
