# Supplementary material for: Multicomponent new particle formation from sulfuric acid, ammonia, and biogenic vapors
Source: Sci Adv. 2018 Dec 12;4(12):eaau5363. doi: 10.1126/sciadv.aau5363 (PMC6291317; doi:10.1126/sciadv.aau5363)
Supplement: http://advances.sciencemag.org/cgi/content/full/4/12/eaau5363/DC1 [file supp_4_12_eaau5363__index.html]

Science Advances | Science Advances

## Supplementary Materials

**This PDF file includes:**

- Supplementary Materials and Methods
- Fig. S1. The effect of different additional vapors on the NPF rates (*J*2.5).
- Fig. S2. The effect of different additional vapors on the biogenic nucleation rate (*J*1.7) at different NO*x* concentrations.
- Fig. S3. Nucleation rates (*J*1.7) as a function of the MT to NO*x* ratio (MT/NO*x*).
- Fig. S4. Nucleation rates (*J*1.7) as a function of NH3 mixing ratio.
- Fig. S5. Modeled versus measured nucleation rates.
- Fig. S6. Modeled versus measured GRs.
- Fig. S7. Positive ions and ion clusters detected during multicomponent NPF in the CLOUD chamber.
- Fig. S8. Global annual mean concentrations of vapors involved in NPF.
- Table S1. Pearson’s correlation coefficient (*R*) between *J*1.7 and the concentration of different precursors in the chamber.
- References (*41*–*56*)

Download PDF

**Files in this Data Supplement:**

- Adobe PDF - aau5363\_SM.pdf
