# Supplementary material for: Multicomponent new particle formation from sulfuric acid, ammonia, and biogenic vapors
Source: Sci Adv. 2018 Dec 12;4(12):eaau5363. doi: 10.1126/sciadv.aau5363 (PMC6291317; doi:10.1126/sciadv.aau5363)
Supplement: http://advances.sciencemag.org/cgi/content/full/4/12/eaau5363/DC1 [file aau5363_SM.pdf]

Science Advances
AAAS

advances.sciencemag.org/cgi/content/full/4/12/eaau5363/DC1

Supplementary Materials for

Multicomponent new particle formation from sulfuric acid, ammonia, and biogenic vapors

Katrianne Lehtipalo*, Chao Yan, Lubna Dada, Federico Bianchi, Mao Xiao, Robert Wagner, Dominik Stolzenburg, Lauri R. Ahonen, Antonio Amorim, Andrea Baccarini, Paulus S. Bauer, Bernhard Baumgartner, Anton Bergen, Anne-Kathrin Bernhammer, Martin Breitenlechner, Sophia Brilke, Angela Buchholz, Stephany Buenrostro Mazon, Dexian Chen, Xuemeng Chen, Antonio Dias, Josef Dommen, Danielle C. Draper, Jonathan Duplissy, Mikael Ehn, Henning Finkenzeller, Lukas Fischer, Carla Frege, Claudia Fuchs, Olga Garmash, Hamish Gordon, Jani Hakala, Xucheng He, Liine Heikkinen, Martin Heinritzi, Johanna C. Helm, Victoria Hofbauer, Christopher R. Hoyle, Tuija Jokinen, Juha Kangasluoma, Veli-Matti Kerminen, Changhyuk Kim, Jasper Kirkby, Jenni Kontkanen, Andreas Kürten, Michael J. Lawler, Huajun Mai, Serge Mathot, Roy L. Mauldin III, Ugo Molteni, Leonid Nichman, Wei Nie, Tuomo Nieminen, Andrea Ojdanic, Antti Onnela, Monica Passananti, Tuukka Petäjä, Felix Piel, Veronika Pospisilova, Lauriane L. J. Quéléver, Matti P. Rissanen, Clémence Rose, Nina Sarnela, Simon Schallhart, Simone Schuchmann, Kamalika Sengupta, Mario Simon, Mikko Sipilä, Christian Tauber, António Tomé, Jasmin Tröstl, Olli Väisänen, Alexander L. Vogel, Rainer Volkamer, Andrea C. Wagner, Mingyi Wang, Lena Weitz, Daniela Wimmer, Penglin Ye, Arttu Ylisirniö, Qiaozhi Zha, Kenneth S. Carslaw, Joachim Curtius, Neil M. Donahue, Richard C. Flagan, Armin Hansel, Ilona Riipinen, Annele Virtanen, Paul M. Winkler, Urs Baltensperger, Markku Kulmala*, Douglas R. Worsnop

*Corresponding author. Email: katrianne.lehtipalo@helsinki.fi (K.L.); markku.kulmala@helsinki.fi (M.K.)

Published 12 December 2018, *Sci. Adv.* **4**, eaau5363 (2018)
DOI: 10.1126/sciadv.aau5363

This PDF file includes:

Supplementary Materials and Methods
Fig. S1. The effect of different additional vapors on the NPF rates ($J_{2.5}$).
Fig. S2. The effect of different additional vapors on the biogenic nucleation rate ($J_{1.7}$) at different NO_x concentrations.
Fig. S3. Nucleation rates ($J_{1.7}$) as a function of the MT to NO_x ratio (MT/NO_x).
Fig. S4. Nucleation rates ($J_{1.7}$) as a function of NH_3 mixing ratio.
Fig. S5. Modeled versus measured nucleation rates.
Fig. S6. Modeled versus measured GRs.
Fig. S7. Positive ions and ion clusters detected during multicomponent NPF in the CLOUD chamber.
Fig. S8. Global annual mean concentrations of vapors involved in NPF.
Table S1. Pearson's correlation coefficient (R) between $J_{1.7}$ and the concentration of different precursors in the chamber.
References (*41–56*)

Supplementary Materials and Methods

Main instrumentation

The particle concentration and number size distribution in the chamber were measured with several independent instruments. The particle size magnifier (PSM(*41*), Airmodus Ltd.), together with a condensation particle counter (CPC) was used to determine the number concentrations of the smallest particles. The PSM uses diethylene glycol as working fluid and achieves supersaturated conditions by turbulently mixing heated saturated air with the sample flow. Particle growth takes place in a cooled growth tube, and in the external CPC, which is also used to count the particles. Since the saturation ratio can be quickly adjusted by altering the flow rate of the saturated air, the cut-off diameter of the PSM can be varied. In this study two PSMs were operated with fixed cut-off sizes and one in scanning mode, which allows determining the particle concentration at several different cut-off sizes (1.7 nm used in this study), as well as the number size distributions between about 1 and 3 nm(*42*). The instruments were calibrated before the campaign using size-selected tungsten oxide particles. Additionally, a conventional butanol ultra-fine CPC (TSI 3776) with a cut-off size of ca. 2.5 nm was used.

The size distribution of particles between 1.7 and 8 nm was measured with a newly developed instrument, the DMA-train(*43*). It consists of six differential mobility analyzers (DMAs) and six CPCs operated in parallel at fixed sizes. This provides high time-resolution and allows exploitation of the full counting statistics at all six sizes. Thereby high sensitivity to low particle concentrations is obtained. For larger particles we used a commercial nano-SMPS (TSI 3938) together with a water-CPC (TSI 3788), and a home-built SMPS, consisting of a TSI X-ray source, a long DMA and a CPC (TSI 3010). The full size distribution produced by combining the different measurements from different instruments thus spanned a range from about 1 to 500 nm.

The ion concentration and size distribution were measured using a neutral cluster and air ion spectrometer (NAIS(*44*), Airel Ltd.). It simultaneously determines the number size distribution of positive and negative ions in the range of 0.75–45 nm mobility diameter with two cylindrical mobility spectrometers in parallel, one for each polarity. Additionally, a corona charger is periodically switched on to charge the aerosol for the detection of the total particle size distribution in the size range of 2–45 nm.

Concentrations of sulfuric acid and highly oxygenated molecules (HOMs) were measured with a nitrate-ion based chemical ionization atmospheric pressure interface time-of-flight mass spectrometer (CI-APi-TOF(*34, 45*)). The nitrate ions are produced by exposing HNO_3 containing sheath flow to an x-ray source. After charging in a drift tube, the sample enters the APi where it gets focused and the pressure is gradually reduced to ca. 10^{-6} mbar. Subsequently, the sample is guided to the TOF region, where the molecules are separated according to their mass-to-charge ratio and detected by a microchannel plate detector. Similar instruments without the chemical charging unit (APi-TOF(*46*)) were used in negative and/or positive mode to detect and identify negative and positive ions and charged clusters.

To quantify sulfuric acid and HOMs, we conducted a calibration and applied corrections similar to our previous work(*15, 32*). Briefly, in separate experiments the OH concentration in the chamber was determined using 1,3,5-trimethylbenzene in CLOUD10 and 1,2,4-trimethylbenzene in CLOUD11. Sulfuric acid production rates were calculated based on the OH and SO_2 concentration in the chamber. Then, the concentration of sulfuric acid in the chamber could be determined using the production rate and losses, including wall loss and condensation loss to aerosol particles. Additional corrections including the instrument transmission correction(*47*), as well as corrections for sampling losses for HOMs were applied. The overall uncertainty in the sulfuric acid and HOMs concentrations is estimated to be ca. 40%. The raw data were analyzed with the MATLAB toftools package(*46*). The elemental composition of each peak was identified using high-resolution peak fitting, based on which we further categorized the HOMs into four different groups: non-nitrate HOM monomer ($C_{4-10}H_xO_y$), non-nitrate dimer ($C_{11-20}H_xO_y$), organonitrate monomer ($C_{4-10}H_xO_yN_{1-2}$), and organonitrate dimer ($C_{11-20}H_xO_yN_{1-2}$). The CI-APi-TOF mainly detects highly oxygenated compounds, with y≥4 for monomers and y≥6 for dimers.

Ammonia (NH_3) concentrations were measured with a quadrupole chemical ionization mass spectrometer (CIMS) equipped with an APi inlet(*48*). Positively charged water clusters ($(H_2O)_n{\cdot}H_3O^+$) were used for the detection of ammonia(*49*). The primary ions are formed by ionizing humidified synthetic air through a corona discharge at ambient pressure(*50*). Neutral ammonia molecules in the sample air interact with the ionized water clusters forming $(H_2O)_nNH_4^+$, which are mainly detected as NH_4^+ since most of the water molecules

evaporate in the collision-dissociation cell of the CIMS. The instrument was calibrated before and after the experiments for the relevant range of NH_3; the calibration curves indicated an excellent linearity and a low detection limit of around 20 pptv. The instrumental background was found to be approximately 100 pptv. The measurements have an estimated overall uncertainty of a factor of two because different inlet systems had to be used between the instrument calibration and the sampling from the CLOUD chamber. For some of the early experiments in CLOUD10 the CIMS was not available, therefore different methods had to be used for deriving the ammonia mixing ratios. Recently, it was reported that ammonia can also be detected in the negative ion mode using $(HNO_3)_nNO_3^-$ primary ions(*51*). The observed $NH_3(HNO_3)_{1,2}NO_3^-$ clusters were used to quantify the ammonia concentrations with the CI-APi-TOF from a cross calibration with the CIMS when both instruments were measuring in parallel during later experiments. However, when only very small amounts of ammonia were added to the CLOUD chamber, the sensitivity of the CI-APi-TOF method was not high enough. Therefore, for those experiments, the mixing ratio was estimated based on the flow of ammonia into the chamber and an experimentally determined wall loss life time(*21*). The overall scale uncertainty of these methods is bracketed by a factor 2.5 towards lower values and a factor of 4 towards higher values. In CLOUD11, NH_3 concentrations were measured with a high-resolution time-of-flight mass spectrometer (H-TOF) using protonated water clusters, and the values at high NH_3 concentration were cross-checked against a commercial PICARRO NH_3 analyzer. The estimated uncertainty due to the calibration methods is ca. ± 50%.

The concentrations of monoterpenes and other volatile organic compounds were measured with a newly developed version of the proton transfer reaction time-of-flight mass spectrometer (PTR-TOF-MS; model: PTR3(*52*)). The PTR3 has a new inlet using center-sampling through a critical orifice reducing wall losses of low volatility compounds. In addition, the new ionization chamber allows a 30-fold longer reaction time and a 40-fold pressure increase compared to standard PTR-TOF-MS instruments. Coupled to the latest quadrupole-interfaced Long-ToF mass analyzer (TOFWERK), sensitivities of up to 20 000 cps/ppbv at a mass resolution of 8000 m/Δm were achieved.

Gas monitors were used to measure the concentration of sulfur dioxide (SO_2, Thermo Fisher Scientific, Inc. 42i-TLE), ozone (O_3, Thermo Environmental Instruments TEI 49C) and water (dew point mirror from EdgeTech). Nitric oxide (NO) concentrations were determined

from a commercially available NO monitor (ECO PHYSICS, model: CLD 780 TR) with a chemi-luminescence detector. The detection limit was ca. 3 pptv with an integrating time of 60 s. During CLOUD10, the amount of nitrogen dioxide (NO_2) was measured with a cavity attenuated phase shift nitrogen dioxide monitor (CAPS NO_2, Aerodyne Research Inc.) at the bottom of the chamber (close to the gas inlet ports). During CLOUD11, additionally a cavity enhanced differential optical absorption spectroscopy (CE-DOAS) instrument was deployed at the level of the sampling ports. The concentrations measured at these two locations generally agreed within 20% for different NO_2 injection rates and UV-light settings, which can be interpreted as an upper limit for the chamber inhomogeneity. The baseline of the instruments was monitored periodically by flushing them with synthetic air.

Determining nucleation and growth rates

The nucleation rates (J) were calculated from the time derivative of the total particle concentration and corrected for the particle losses in the chamber using the full size distribution

$$J = \frac{dN}{dt} + S_{dil} + S_{wall} + S_{coag} \ (\mathrm{cm^{-3}\,s^{-1}}) \quad (1)$$

where N is the particle number concentration above a certain cut-off size (d_p) to which the nucleation rate is calculated. The dilution correction S_{dil} arises from the fact that the chamber is constantly flushed with synthetic air to account for the instruments' sample flows

$$S_{dil} = N \cdot k_{dil} \ (\mathrm{cm^{-3}\,s^{-1}}), \quad (2)$$

where $k_{dil} = 1.437 \cdot 10^{-4}\ \mathrm{s^{-1}}$ for CLOUD 10 and $1.58 \cdot 10^{-4}\ \mathrm{s^{-1}}$ for CLOUD11.

Diffusional losses to the chamber walls (S_{wall}) were determined empirically by observing the decay of the sulfuric acid monomer concentration in the chamber. The wall loss rate is inversely proportional to the particle size

$$k_{wall}(d_p', T) = 2.116 \cdot 10^{-3} \cdot \left(\frac{T}{T_{ref}}\right)^{0.875} \cdot \left(\frac{d_{p,ref}}{d_p'}\right) \quad (\mathrm{s^{-1}}) \quad (3)$$

where d_p' is the mobility diameter of the particle, $d_{p,ref}$ is the mobility diameter of the sulfuric acid monomer (= 0.82 nm), T_{ref} = 278 K, and T is the actual chamber temperature. Thus the

total wall loss for particles larger than d_p is

$$S_{wall}(d_p,T) = \sum_{d_p'=d_p}^{d_{p,max}'} N(d_p') \cdot k_{wall}(d_p',T)\ (\mathrm{cm^{-3}\,s^{-1}}) \tag{4}$$

Coagulation losses to the surface of larger aerosol particles (S_{coag}) were calculated from the measured number size distribution of particles present in the chamber

$$S_{coag}(d_{p,k}) = \sum_{d_{p,i}=d_{p,k}}^{d_{p,max}} \sum_{d_{p,j}=d_{p,i}}^{d_{p,max}} \delta_{i,j} \cdot K(d_{p,i}, d_{p,j}) \cdot N_i \cdot N_j\ (\mathrm{cm^{-3}\,s^{-1}}) \tag{5}$$

where $K(d_{p,i},d_{p,j})$ is the coagulation coefficient for particles of size $d_{p,i}$ and $d_{p,j}$, N_i and N_j are the number densities of particles in a size bins i and j, and $\delta_{i,j} = 0.5$, if i = j and $\delta_{i,j} = 1$, if i ≠ j.

The nucleation rates at 1.7 nm ($J_{1.7}$) were calculated from the scanning PSM and verified against the values calculated from the two other PSMs and the butanol CPC at fixed cut-off sizes. It should be noted that there is an uncertainty of about 0.5 nm in the cut-off size of the particle counters due to the effect of composition and charge on the detection efficiency(*53*). To account for this, we verified the cut-off size of the PSM for each chemical system in the chamber by comparing the concentration and rising time of the PSM at different saturator flow rates against the different size bins of the NAIS, which has been shown to be very accurate in determining the ion mobility(*54*). The *J* value given for each experiment is the median value after reaching stable conditions. The uncertainty in the nucleation rates (given as error bars in the figures) was calculated with error propagation method, taking into account both the systematic and statistical errors and run-to-run repeatability. The systematic errors include errors on concentration measurement (10%), dilution (10%), and wall loss (20%). The statistical errors include uncertainty on *dN/dt* and coagulation sink, which varied from run to run depending on the stability of the measurement conditions. The run-to-run repeatability of *J* in CLOUD under nominally identical conditions is ca. 30%.

The growth rates were calculated using the appearance time method(*31, 42*) from the scanning PSM (1-3 nm), the DMA-train (2-3 nm and 3-8 nm) and the nano-SMPS (7-25 nm). The error in GR was estimated from the 95% confidence intervals for the (d_p, time) – fits, which were used to determine the GRs. The appearance times from the different

instruments were also checked for consistency in the overlapping size regions. While the appearance time method is simple to apply for different instruments, and provides a useful estimation of particle growth, it should be kept in mind that the growth rates especially in the smallest size ranges are difficult to define, and different methods might differ from each other depending non-linearly on the environmental conditions(*55*, *56*).

Supplementary figures

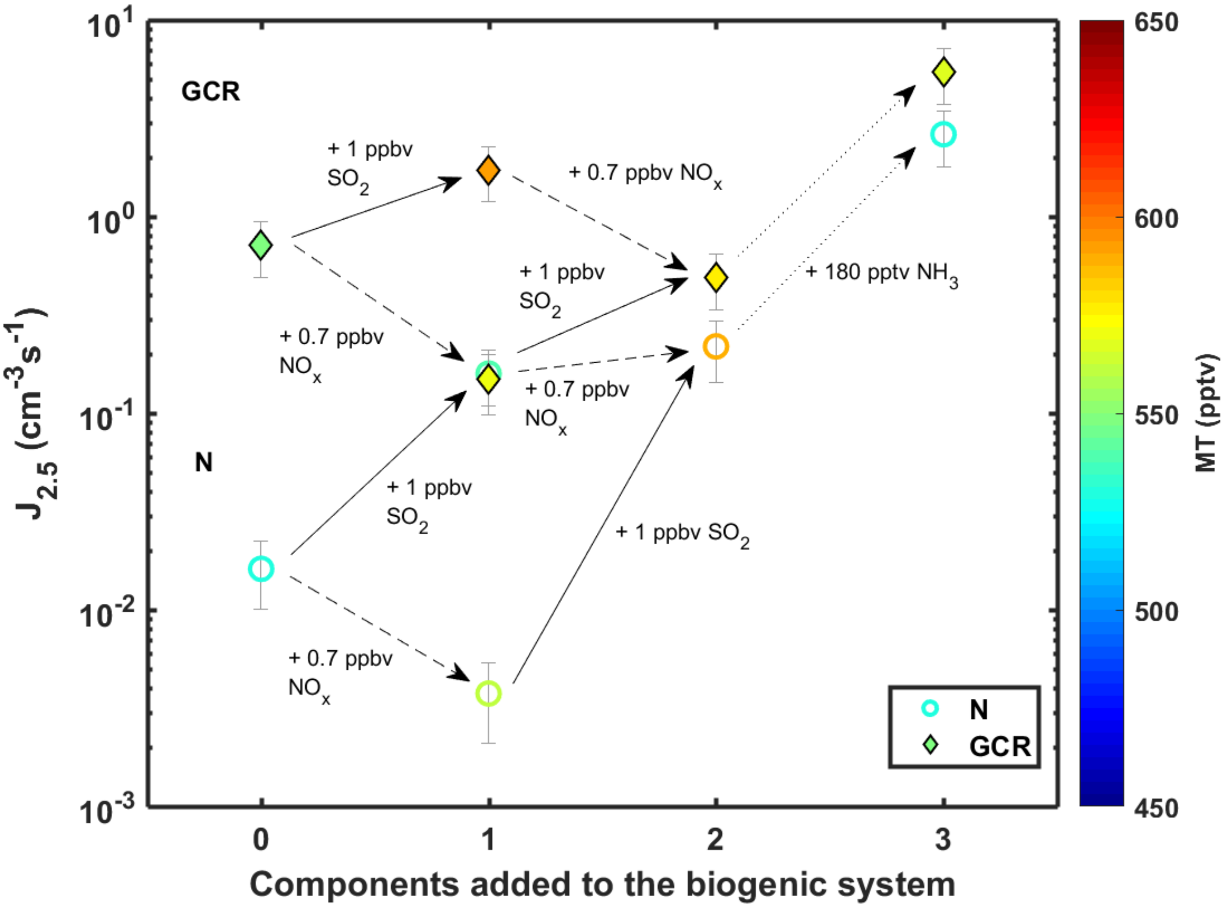

Fig. S1. The effect of different additional vapors on the NPF rates ($J_{2.5}$). All points have similar monoterpene (530-590 pptv) and ozone (40 ppbv) mixing ratios. The leftmost points were measured with only monoterpenes added to the chamber, and each step to the right represents addition of one more component to the system. Solid arrows describe the addition of ca. 1 ppbv SO_2 (resulting in an H_2SO_4 concentration of $1\text{-}2\cdot10^7$ cm^{-3}), dashed arrows the addition of ca. 0.7 ppbv NO_x and dotted arrows the addition of ca. 180 pptv NH_3. Circles are experiments at neutral (N) and diamonds at GCR conditions. Colors of the symbols indicate the measured monoterpene mixing ratio. See Fig. 1 for the formation rate of 1.7 nm particles.

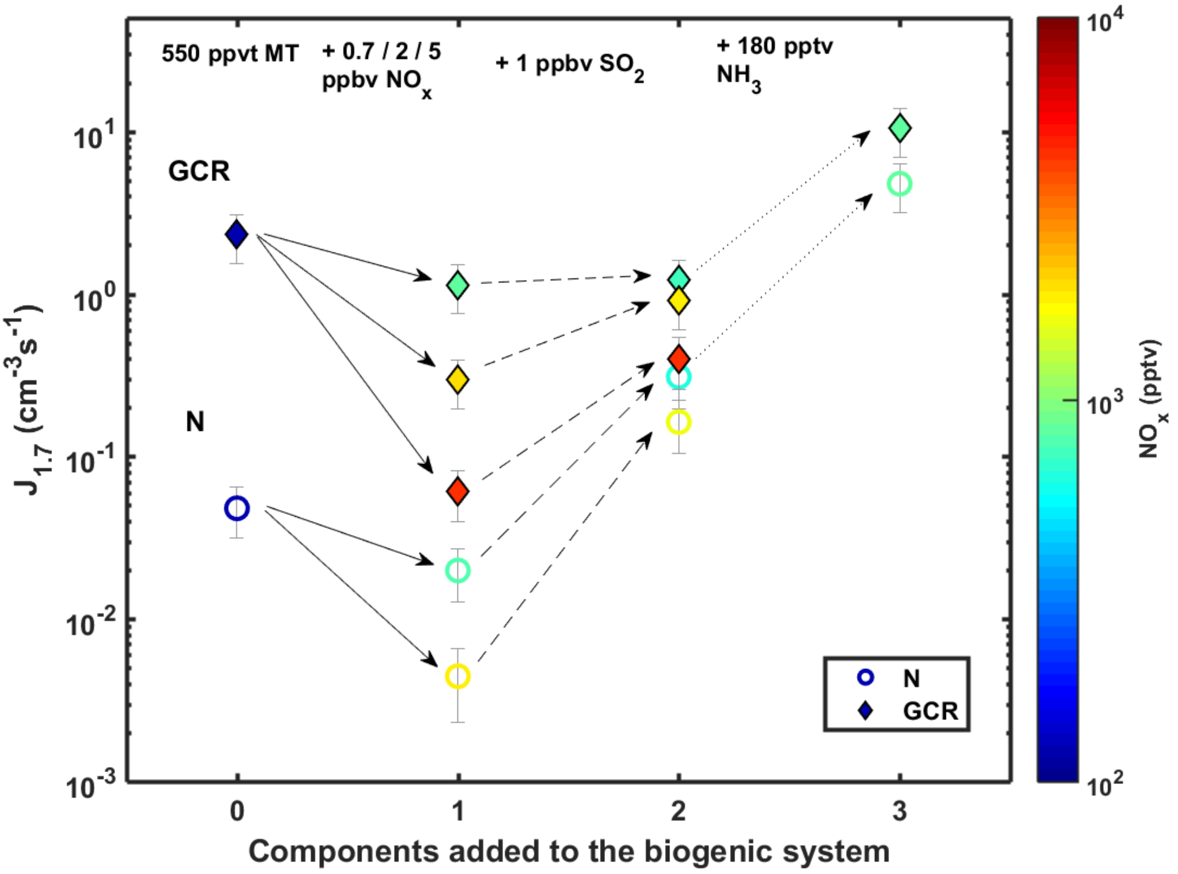

Fig. S2. The effect of different additional vapors on the biogenic nucleation rate ($J_{1.7}$) at different NO_x concentrations. All points have a similar monoterpene mixing ratio (500-590 pptv). The leftmost points were measured with only monoterpenes added to the chamber, and each step to the right represents the addition of one more component to the system. The solid arrows describe the addition of NO_x (~ 0.7, 2 or 5 ppbv), the dashed arrows the addition of ~1 ppbv SO_2 (resulting in an H_2SO_4 concentration of $1\text{-}2{\cdot}10^7$ cm^{-3}) and dotted arrows the addition of ~180 pptv NH_3. Circles are experiments at neutral and diamonds at GCR conditions. The color of the symbol indicates the measured NO_x concentration.

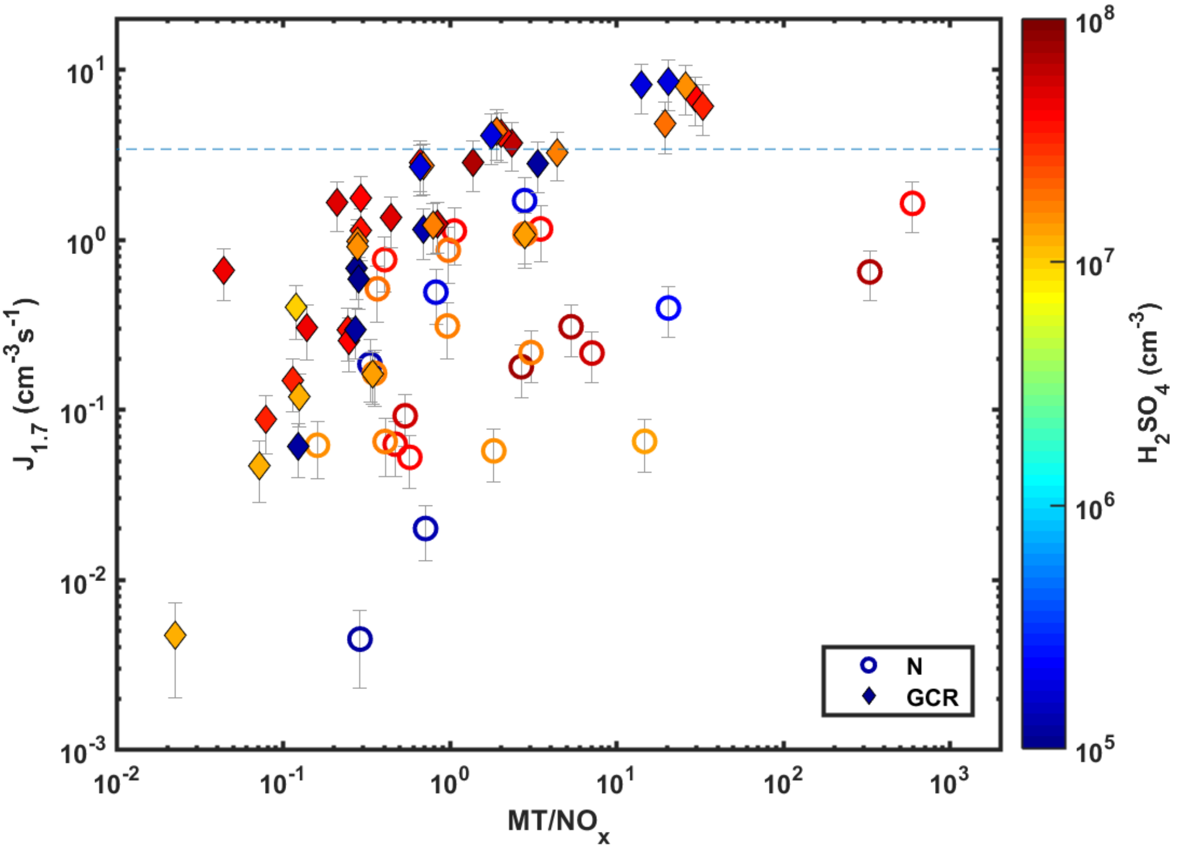

Fig. S3. Nucleation rates ($J_{1.7}$) as a function of the MT to NO$_x$ ratio (MT/NO$_x$). All experiments were performed without added NH_3 at a constant NO/NO_2 ratio of ca. 0.6%. The color indicates the sulfuric acid concentration. The blue points (lowest H_2SO_4) were measured without SO_2 added to the chamber (pure biogenic nucleation). The dashed line gives the maximum rate from ion-induced nucleation, based on the ion pair production rate in CLOUD under GCR conditions[14].

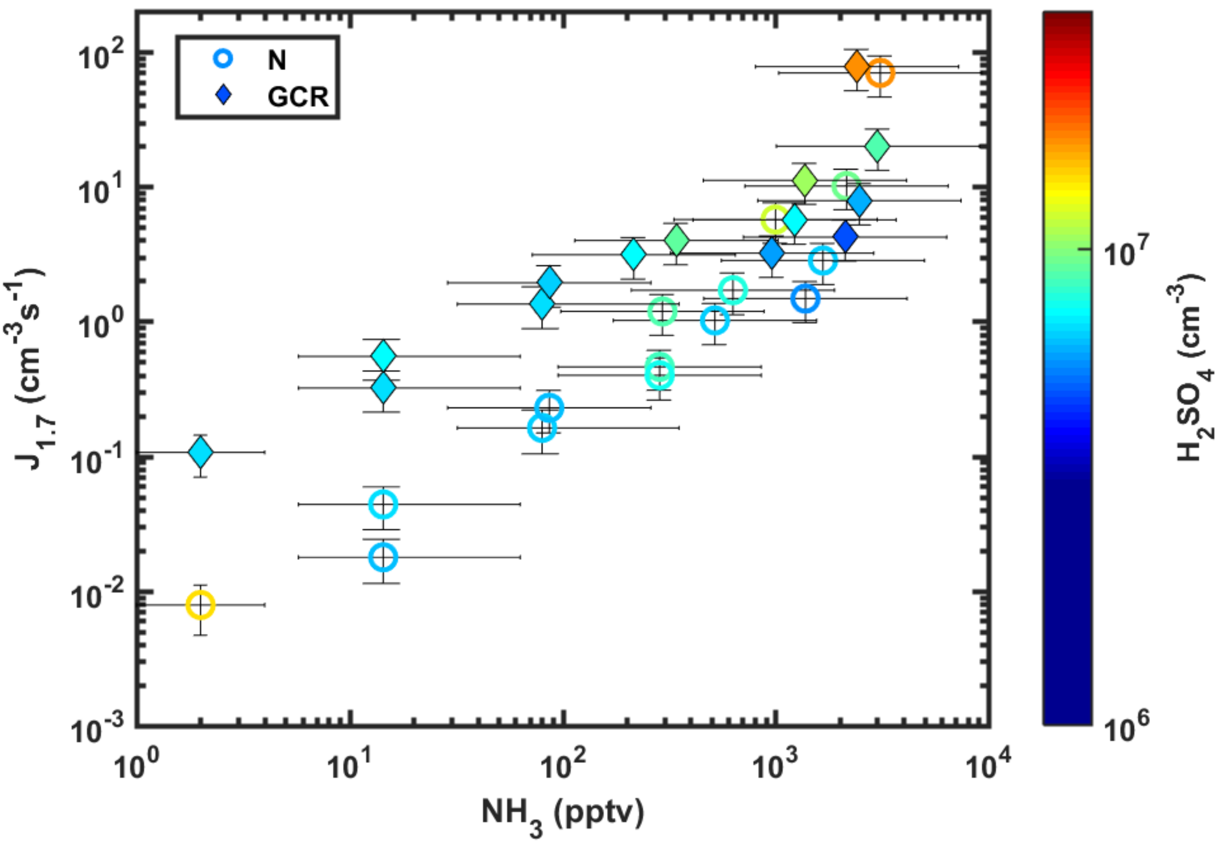

Fig. S4. Nucleation rates ($J_{1.7}$) as a function of NH_3 mixing ratio. Open circles refer to neutral experiments, closed diamonds to GCR experiments, and the color refers to the H_2SO_4 concentration. All points were measured at 278 K and 38% RH with constant MT (ca. 250 pptv) and NO_x (ca. 2 ppbv) mixing ratios. Due to the unavailability of the CIMS to measure NH_3 in this set of experiments, the lowest NH_3 values (<200 pptv) were estimated from the NH_3 flow to the chamber, while the values larger than 200 pptv were derived from the CI-APi-TOF (see Materials and Methods).

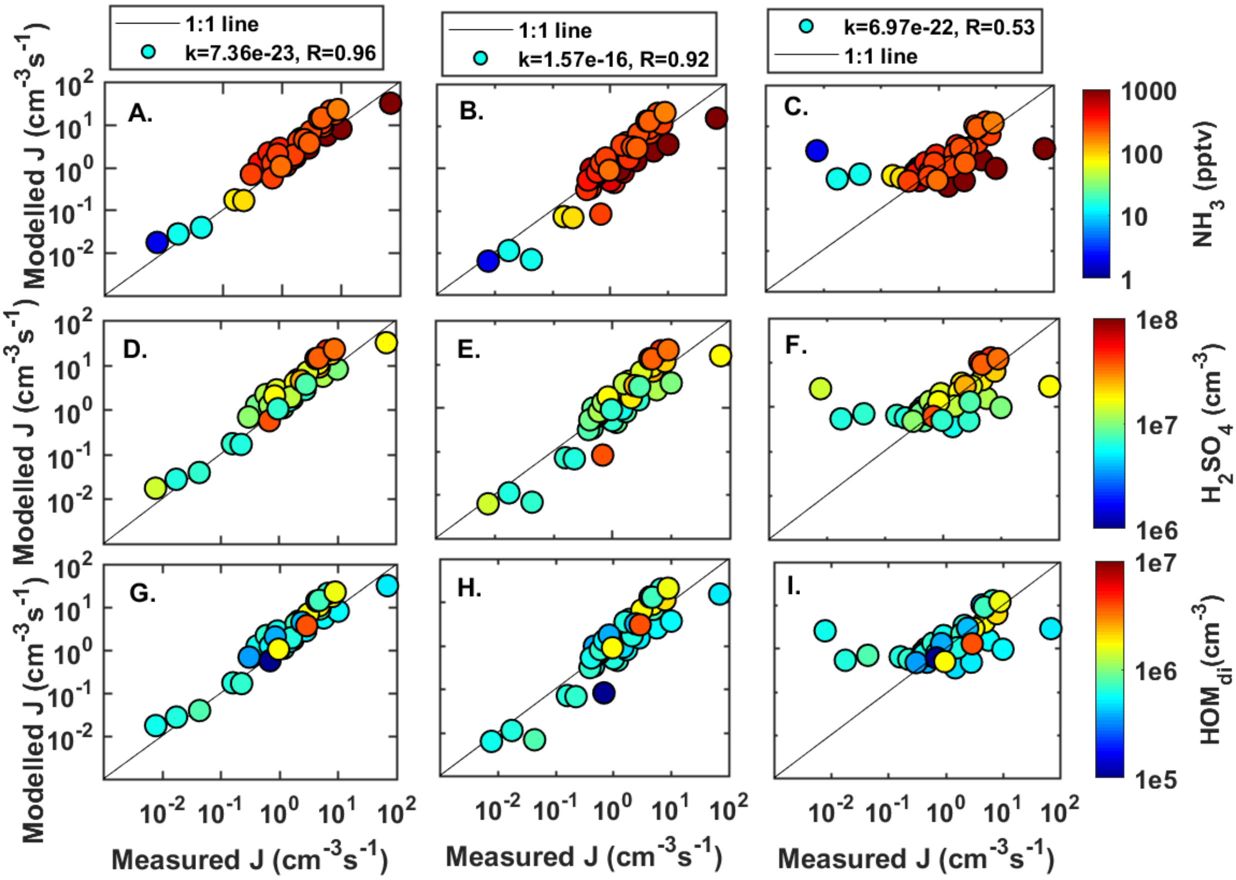

Fig. S5. Modeled versus measured nucleation rates. (**A**, **D**, **G**) modelled nucleation rates using equation (1), (**B**, **E**, **H**) modelled nucleation rates using equation (1) with [MT/NO_x] in place of [HOM_{di}] and (**C**, **F**, **I**) modelled nucleation rates using an earlier CLOUD parametrization(*11*) without NH_3 dependency, assuming [BioOxOrg]=[HOM]. The data were colored either by the NH_3 (A-C), H_2SO_4 (D-F) or non-nitrate HOM dimer **(G-I)** concentration. Only neutral experiments are presented for clarity. *R* is Pearson's correlation coefficient between $\log_{10}(J_{measured})$ and $\log_{10}(J_{modelled})$.

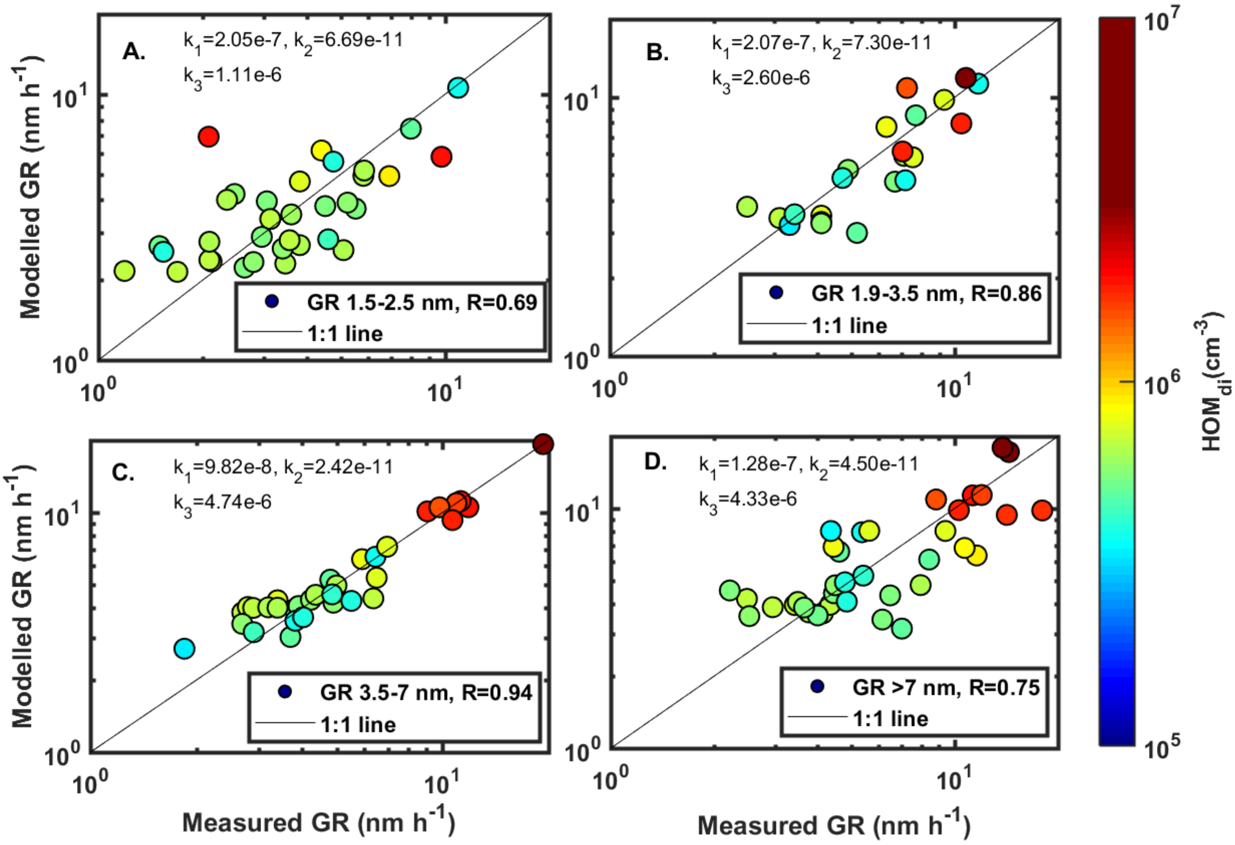

Fig. S6. Modeled versus measured GRs. The modelled growth rates were calculated using equation (4) separately for 4 different size ranges (**A-D**). The data points are colored by the non-nitrate HOM dimer concentration. It can be seen from the values of the free parameters k_1-k_3 that the importance of sulfuric acid decreases and the importance of organics increases towards larger sizes. For particles larger than 7 nm a different set of organics should probably be considered, as the correlation coefficient R between measured and modelled GRs starts to decrease. GR in the size range 1.5-2.5 nm was determined from the PSM, 1.9-3.5 nm and 3.5-7 nm from the DMA-train and >7 nm from the nano-SMPS (see Materials and Methods). *R* is Pearson's correlation coefficient between $\log_{10}(GR_{measured})$ and $\log_{10}(GR_{modelled})$.

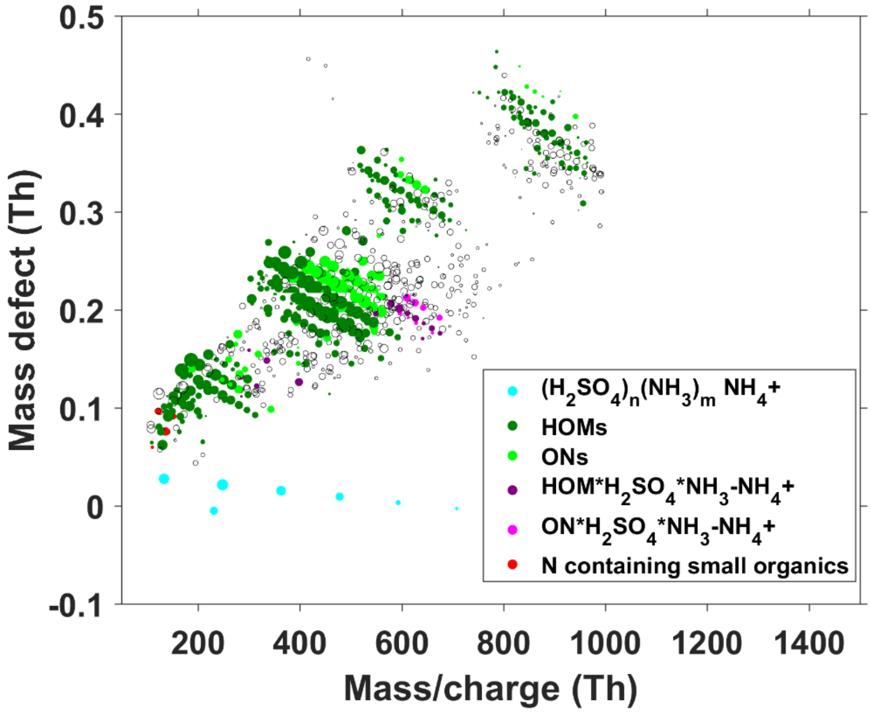

Fig. S7. Positive ions and ion clusters detected during multicomponent NPF in the CLOUD chamber. The mass defect shows the difference between nominal and exact mass of the ions detected with the positive APi-TOF during experiments with ca. 600/1200 pptv MT, 1-2·10^7 cm^{-3} of H_2SO_4, 1 ppbv of NO_x (ca. 70 pptv of NO) and 200 pptv of NH_3 in the chamber. Colored symbols are identified ions (see legend) and open symbols are unidentified ions. The symbol size corresponds to the relative signal intensity on a logarithmic scale.

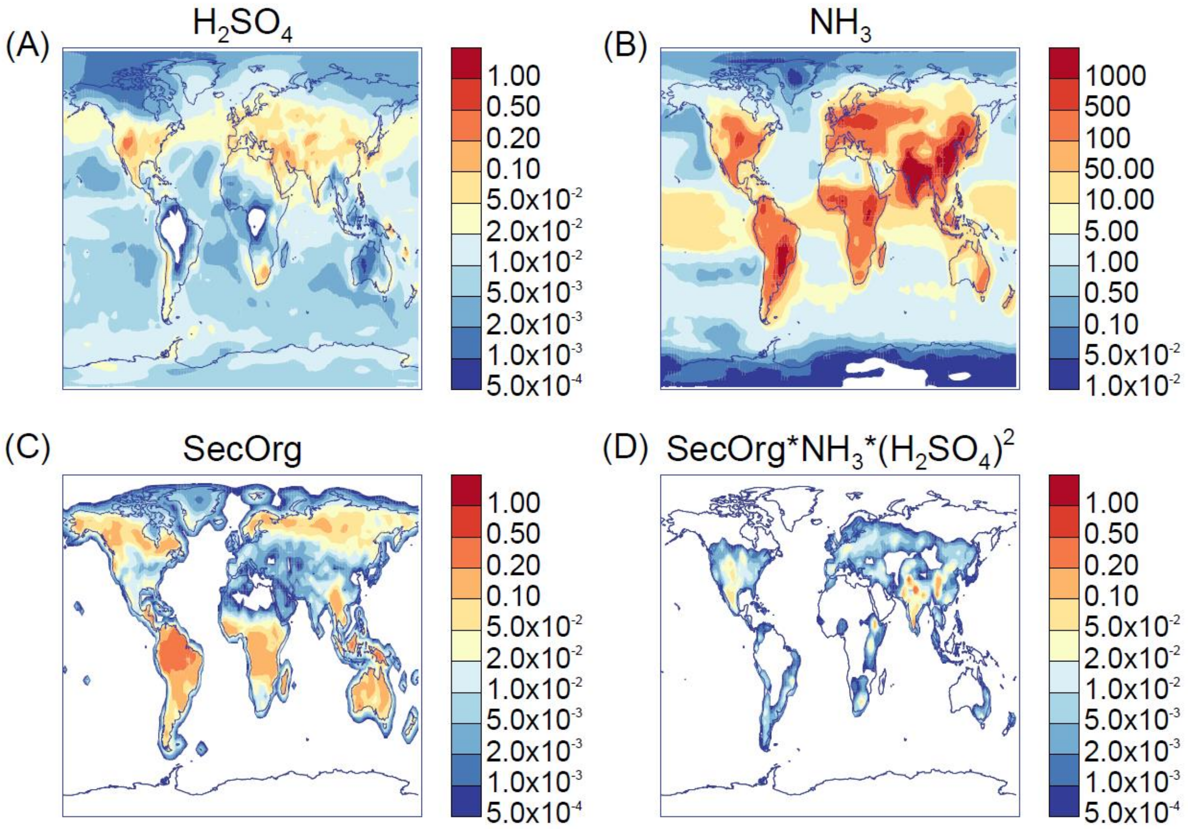

Fig. S8. Global annual mean concentrations of vapors involved in NPF. The colors indicate the mixing ratios in pptv, at approximately 500 m above the surface (cloud base level), from the TOMCAT chemical transport model[32] with the embedded GLOMAP aerosol model[33]. (**A**) shows sulfuric acid, (**B**) ammonia and (**C**) the gas `SecOrg', which is a proxy for low volatility organic vapors produced from monoterpenes. The spatial distribution of SecOrg matches approximately the distribution of biogenic HOMs in the atmosphere. Panel (**D**) shows the overlap of the vapor concentrations, following the functional form of eq. (1).

Table S1. Pearson's correlation coefficient (*R*) between $J_{1.7}$ and the concentration of different precursors in the chamber. The data set from CLOUD10 was divided into experiments with and without NH_3, and into neutral (N) and GCR conditions. *R* is the correlation coefficient, and *p* its statistical significance level (0.05 means a 5% chance of getting the given correlation by chance). Coefficients higher than 0.75 are colored red. The NaN values are due to missing ammonia measurements during the first part of the experiment, when no NH_3 was added.

**Correlation coefficient with $J_{1.7}$**	**No NH_3 GCR**		**No NH_3 N**		**NH_3 GCR**		**NH_3 N**	
Nr of data points	41		28		30		40	
	R	*p*	***R***	*p*	***R***	*p*	***R***	p
H_2SO_4	**-0.06**	0.69	**0.04**	0.85	**0.22**	0.24	**0.14**	0.38
MT	**0.60**	<0.01	**0.79**	<0.01	**0.02**	0.90	**0.03**	0.84
NO_x	**-0.50**	<0.01	**-0.07**	0.74	**0.03**	0.88	**0.05**	0.76
MT/NO_x	**0.81**	<0.01	**0.46**	0.02	**0.00**	0.98	**0.00**	0.98
HOM monomers	**0.08**	0.61	**0.61**	<0.01	**-0.10**	0.60	**0.02**	0.89
HOM dimers	**0.86**	<0.01	**0.82**	<0.01	**-0.10**	0.61	**-0.02**	0.89
Non-nitrate HOM monomers	**0.63**	<0.01	**0.82**	<0.01	**-0.09**	0.65	**0.03**	0.86
Nitrate HOM monomers	**-0.42**	0.01	**0.01**	0.94	**-0.07**	0.73	**0.01**	0.97
Non-nitrate HOM dimers	**0.97**	<0.01	**0.83**	<0.01	**-0.04**	0.84	**0.00**	1.00
Nitrate HOM dimers	**-0.34**	0.03	**-0.10**	0.63	**-0.28**	0.13	**-0.08**	0.63
Total HOMs	**0.17**	0.28	**0.65**	<0.01	**-0.11**	0.58	**0.02**	0.92
Total non-nitrate HOMs	**0.72**	<0.01	**0.85**	<0.01	**-0.08**	0.68	**0.02**	0.88
Total nitrate HOMs	**-0.42**	0.01	**0.00**	0.99	**-0.09**	0.65	**0.00**	0.99
H_2SO_4 * HOM_{di}	**0.45**	<0.01	**0.44**	0.02	**0.12**	0.54	**0.12**	0.45
H_2SO_4 * HOM_{di} * NH_3	**NaN**	NaN	**NaN**	NaN	**0.82**	<0.01	**0.89**	<0.01
